# Effects of resisted sprint training with ball on speed and agility performance in U-19 elite soccer players

**DOI:** 10.1371/journal.pone.0311002

**Published:** 2024-10-02

**Authors:** Alejandro Sal-de-Rellán, Mehdi Ben Brahim, Ariadna Hernaiz-Sánchez, Raghad Tarwneh, Víctor Martín

**Affiliations:** 1 Faculty of Social Sciences and Communication, Universidad Europea de Madrid, Madrid, Spain; 2 Health and Physical Education Department, Prince Sultan University, Riyadh, Kingdom of Saudi Arabia; 3 Faculty of Health Sciences, Universidad Isabel I, Burgos, Spain; Università degli Studi di Milano: Universita degli Studi di Milano, ITALY

## Abstract

The purpose of this study was to analyze the effects on speed and agility of including ball driving during resisted sprint training in relation to regular soccer training. Thirty male soccer players (age: 18.10 ± 0.66 years; height: 179 ± 0.06 cm; body mass: 76.22 ± 4.76 kg; systematic practice: 8.6 ± 1.73 years) participated in the study and were randomly assigned to an experimental group (EG; n = 18) or a control group (CG; n = 12). The research was conducted during a training camp. The intervention period lasted 6 weeks and during that period all players performed their usual soccer training. However, the EG performed two weekly sessions of a supplementary training of resisted sprints with a ball. Within-group analysis showed significant improvements in 5-m (*p* = 0.005) and 10-m (*p* = 0.016) sprint performance; and New multi-change of direction agility test (NMAT; *p* = 0.002), Illinois (IAT; *p* = 0.002), T-test (*p* = 0.003), Arrowhead COD (Arrowhead-R, *p* = < 0.001; Arrowhead-L, *p* = < 0.001) test and Zigzag agility test (Zigzag-B; *p* = 0.006) from pretest to posttest in EG. However, the CG didn’t show any significant improvements. Between-groups analysis revealed differences in favor of the EG in Zigzag-B, IAT, Arrowhead-R, Arrowhead-L and NMAT. This study’s results support the efficacy of a short-term training program that includes resisted sprints exercises to improve the performance of soccer players.

## Introduction

The improvement of training process should start from the understanding of those physiological and physical demands associated with competition [1]. Previous studies have shown that during the 90 minutes of a soccer match, elite players cover total distance of 10–13 km [2]. Although total distance is an interesting data, the speed associated with these movements seems to be the key to performance [3]. During sprinting, players might cover 185–190 m and reach a maximum velocity of 31 km•h^-1^ [4]. Thus, straight sprinting is the most frequent action in goal situations [5]; the competition demands more and more of these actions [6], and it determines the competitive level of the soccer players [3]. In addition to sprint actions, soccer players perform hundreds of accelerations, decelerations and changes of direction (COD) during the match [7]. Consequently, these actions, as sprinting, should be taken into account when assessing and training soccer players.

By examining the relationship between sprint and performance in soccer players, several strength-training methods have been used to improve sprint, including resistance, plyometric, contrast, eccentric overload and resisted sprint trainings. In this sense, sprinting consists of distinct phases (i.e., the start, acceleration, maximum speed, and deceleration) [8], in which muscle strength and movement technique are fundamental [9]. Therefore, training methods that combine specific motor tasks and high strength demands may be interesting for enhancing sprinting performance of soccer players. In this way, strength-specific exercises have been incorporated into sprint training routines. They are known as resisted sprint training [10]. Resisted sprint training (RST), which involves the soccer player sprinting with an added load, is a type of sprinting training created to increase the neuromuscular activation and enhance recruitment of fast-twitch fibers [11], without substantial changes in running techniques [12]. Depending on the material used, there are different RST modalities that coaches frequently use, such as: sled, weighted vest, parachute, uphill running, elastic cord and partner-resisted drills [13]. Recently, researchers have focused on resisted sled sprinting, specifically sled pulling, as a popular and effective method of sprint training. For instance, in order to analyze the effects of three different training methods (i.e., full-back squat training, RST with sled (12.6% of body mass) and plyometric training) young soccer players were assessed after 8 weeks training [14]. It was observed that focusing improvements on the final phase of the sprint, the RST could be a beneficial method. Along the lines of this research, positive effects have been found with other means of training, such as weighted vests, elastic cords or parachutes [13]. However, it should be noted that negative effects have also been found [15]. These discrepancies in the literature are likely due to many factors, such as: equipment utilized, training load, load direction (i.e., vertical or horizontal) or periodization of training.

There is evidence to support that RST seems effective in enhancing acceleration demands [16]. As the ability to accelerate may positively impact the change of direction speed it is possible to speculate that resisted sprint training may be beneficial [17]. Although the ability to change direction and agility are not the same variable, it should be kept in mind that the former is one of the important factors that contribute to agility [18]. Consequently, the training sessions that aim to improve the change of direction will also enhance agility. In this regard, few studies have assessed agility after a resisted sprints training in soccer players [14,19,20]. As a result, the findings should be interpreted carefully.

To the best of our knowledge, no previous research has included ball driving during the performance of resisted sprints. This fact makes the exercise more complex, but also more specific, since during the game players perform high-intensity efforts (i.e., sprint and change of direction) with the ball [21]. Furthermore, it has been shown that including the ball in conditional exercises for young soccer players improves both conditional performance and technical skills [22]. In this sense, adding a ball to this type of exercise (i.e., resisted sprints) would set it apart from previous research [19,23,24], which found improvements in soccer players’ performance. Therefore, the purpose of this study was to analyze the effects of including ball driving during RST on speed and agility in relation to regular soccer training. Based on the findings of previous research, it was hypothesized that the use of RST would provide greater training overload and improve performance to a greater extent than standard soccer training. In addition, as a novelty, performing ball driving during RST would improve technical skills in ball agility tests.

## Materials and methods

### Participants

Thirty male soccer players (age: 18.10 ± 0.66 years; height: 179 ± 0.06 cm; body mass: 76.22 ± 4.76 kgs; systematic practice: 8.6 ± 1.73 years) from the Tunisian national U-19 team volunteered to participate in this study. They, who regularly competed in national and international competitions, were randomly assigned to either the experimental group (EG, n = 18) or control group (CG, n = 12). An a priori power analysis (G*Power, v3.1.9.2; Universität Kiel, Kiel, Germany) indicated that a sample size of at least 15 was required to achieve power (1- *β*) of 0.80 with an effect size (ES) of 0.30 (moderate effect) and alpha of 0.05. Inclusion criteria were no injuries in the past six months, limited sports participation for over seven days, and having participated in 90% of the training sessions. Players and parents/guards were informed of the study’s procedures, potential risks/benefits, and both signed a written informed consent before starting the investigation. In addition, the study was performed in accordance with the Declaration of Helsinki (2013) and approved by the ethics committee of the University (Ui1-PI080).

### Design

The investigation was conducted during a training camp. The training program was conducted in addition to the regular training sessions, twice a week for six weeks (from 20/11/2023 to 14/1/2024). During the week preceding the experiment, players familiarized themselves with the testing protocols. At the beginning and the end of this intervention, 7 tests were performed: linear speed test, new multi-change of direction agility test (NMAT), Illinois agility test (IAT), T-test, arrowhead COD test, 15-m ball dribbling agility test, and zigzag agility test. These were performed in three nonconsecutive days (i.e., 48-h between testing days). On the first day, players performed the linear speed test, NMAT, and IAT; on the second day, they performed the T-test and arrowhead COD test, and on the third day, ball handling tests were performed: a 15-m ball dribbling agility test and zigzag agility test. Between each evaluation test there was a 5-min of passive recovery. All testing sessions were conducted outdoors, on natural grass, at the same habitual training time (i.e., 10:00–12:00 AM) and under similar environmental conditions (23–25°C), with the same sports clothes, and by the same testers. Before testing, a general and specific warm-up routine was performed, involving 3-min of jogging, followed by 5-min of dynamic and ballistic stretching, and 7-min of progressive sprints and accelerations [25].

During the experimental period, both groups (EG and CG) followed a weekly standard training program. A typical microcycle consists of a recovery, strength, endurance, speed and activation sessions (Table 1). Additionally, there was one soccer match played at the weekend. These training sessions were conducted with the same coach’s instructions. To reduce the influence of uncontrolled variables, players were instructed to maintain their habitual lifestyle and normal dietary intake before and during the study.

**Table 1:** Typical weekly training program during the 6 weeks of intervention.

Monday	Tuesday	Wednesday	Thursday	Friday	Saturday	Sunday
Recovery (75 min) Warm-up: 15 min 60 min Starters Aerobic endurance: 15 min Injury prevention: 30 min Stretching 15 min Nonstarters Injury prevention: 15 min Small-sided games: 30 min Speed endurance:15 m	Rest	Strength (80 min) Warm-up: 15 min 15 min EG: RST (resisted sprint training). CG: Injury prevention. Positional games: 20 min Small-sided games: 30 min	Endurance (90 min) Warm-up: 15 min Positional games: 20 min Large-sided game: 20 min Simulated game: 35 min	Speed (70 min) Warm-up: 15 min 15 min EG: RST (resisted sprint training). CG: Injury prevention. Medium-sided game: 20 min Tactical drills: 20 min	Activation (60 min) Warm-up: 15 min Activation exercises: 10 min Tactical drills: 20 min Strategy: 15 min	Match

### Training intervention

During the training intervention EG performed two-weekly sessions of resisting sprint training (RST), while CG performed injury prevention training. During RST, 1 set and 6 repetitions were performed in each training session. All repetitions were performed with ball conduction (Table 2). A combined-resisted sprint approach was employed, involving both a weighted vest and a sled. In the first and second repetition, a weighted vest with a load equivalent to 13% of body weight was used [23]. In the third and fourth repetition, the same load was applied, but a sled was used as resistance. In the fifth and sixth repetition, players wore a weighted vest at 6.5% while pulling a sled with a load at 6.5% of body weight during sprints. The training volume progressed from the first to the fourth week (i.e., from 120 m to 300 m) and was reduced in the last two weeks to achieve a tapering effect. Participants were encouraged to execute all sprints at their maximum speed. During the injury prevention training, the players performed a set of 6 exercises: Nordic Hamstring, Sidestepping (X-Band), SL Deadlift, Side Plank Clam, Split-Squat and Bird-Dog [26]. The training volume progressed from the first to the fourth week (i.e., week 1–2 = 6 reps, week 3–4 = 8 reps) and was reduced in the last two weeks (week 5–6 = 6 reps). The total intervention time for the two groups (EG and CG) was the same (15 min).

**Table 2:** Summary of training load in the intervention period by EG.

Weeks	Distance per repetition (m)	Set	Rep	Total distance per training (m)	Total distance per week (m)	Recovery between repetitions (sec)	Exercises^1^
1	20	1	6	120	240	60	1^st^ and 2^nd^ repetition with a weight vest of 13% body mass. 3^rd^ and 4^th^ repetition with a pulling a sled of 13% body mass. 5^th^ and 6^th^ repetition with a weight vest of and pulling a sled (each 6.5% of body mass).
2	30	1	6	180	360	60	
3	40	1	6	240	480	60	
4	50	1	6	300	600	60	
5	40	1	6	240	480	60	
6	30	1	6	180	360	60	

^1^ Exercises were always the same in all weeks.

### Performance tests

#### Linear speed test

Players started from a standing position, 0.5-m behind the first set of infrared photoelectric cells (Cell Kit Speed Brower, USA), before running at maximal speed to the second infrared photoelectric cell. Participants were encouraged to execute two maximum sprints of 30-m, with 5-m and 10-m split times, allowing a 2-min of passive recovery between trials. The fastest time was retained for analysis. The intraclass correlation coefficients (ICCs) and the coefficients of variation (CVs) for the 30-m were ICC = .97, CV = 1.4%; 10-m ICC = .95, CV = .9%; 5-m ICC = .94, CV = .7%.

#### New multi-change of direction agility test (NMAT)

This test has been described in the literature by Brahim et al. [27]. Using the same set of infrared photoelectric cells (Cell Kit Speed Brower, USA), players’ speeds during a 25-m agility run were measured. Two trials were performed with a 5-min of passive recovery. The fastest time of the two attempts was recorded. ICC and CV for NMAT was .92 and 1.8%, respectively.

#### Illinois agility test

Illinois Agility Test (IAT) was conducted using a standardized version from previous literature [28]. The test is established with four cones used to indicate the start and two turning points, and four more cones positioned further down toward the start line at equal 3.3-m intervals. The players sprint 10-m, turn and return back to the start line, and then swerve in and out of the four markers, finishing the test with two 10-m sprints in the opposite direction. IAT performance was recorded using an infrared photoelectric cell (Cell Kit Speed Brower, USA). Two attempts were performed with a passive recovery period of 5-min. The best time to complete each trial was recorded in seconds and it was used for the analysis. The ICC and the CV for IAT were .96 and 1.2%, respectively.

**T-test.** T-test was administered using the protocol, as previously outlined by Negra et al. [29]. Its score was recorded as the best time of two attempts, with a 5-min of passive recovery between each. It was assessed with an infrared photoelectric cell (Cell Kit Speed Brower, USA). The ICC and the CV for T-test were .89 and 2.0%, respectively.

#### Arrowhead COD test

Arrowhead COD test is a soccer-specific assessment [30], therefore it was also included. Players took up a sprint starting position behind the starting line. Each participant ran as fast as possible from the start to the middle marks, turned to sprint through the side marks, through the further marks, and back through the start/finish line. Participants completed four trials, two to the left and two to the right, with 5-min of passive recovery between them. The time was recorded in seconds (Cell Kit Speed Brower, USA), and the fastest time for the left side and for the right side were recorded. The ICC and the CV for Arrowhead COD test were .90 and 1.7%, respectively.

#### 15-m ball dribbling agility test

This test was performed under the protocol previously published by Mujika et al. [31]. Infrared photoelectric cells (Cell Kit Speed Brower, USA) were used to record the time spent covering the test. Each player performed two maximal repetitions with 3-min of passive recovery. During the test, players were required to drive a ball. After the slalom section, the ball was kicked under the hurdle while the player cleared it. Players then freely kicked the ball towards either of two small goals placed diagonally 7-m on the left and the right sides of the hurdle, and sprinted to the finish line. The fastest repetition was selected for further analysis. Reliability values for 15-m ball dribbling agility test were ICC = .74, CV = 2.6%.

#### Zigzag agility test

Participants performed a zig-zag agility test with the ball. It consisted of four 5-m sections marked with set at 100° angles [32]. Starting from a standing position with the front foot placed 0.5-m behind the first pair of infrared photoelectric cells (Cell Kit Speed Brower, USA), the players ran and changed direction as quickly as possible with the ball, until they crossed the second pair of photocells, placed 20-m from the starting line. Two repetitions were performed with a 3-min of passive recovery. The fastest time from the two attempts was retained for analysis. Reliability values for Zigzag agility test were ICC = .84, CV = 2.2%.

### Statistical analysis

The descriptive analysis was presented as mean ± standard deviation (SD). For the assumption of normality, the Shapiro-Wilk test was used, which confirmed that the data had a normal distribution, while the Levene test showed that the variance was homogeneous. All variables were normally distributed. A Paired-samples *t*-test was used to evaluate within-group differences, and an analysis of covariance (ANCOVA) was performed to detect possible between-group differences (i.e., EG vs CG), assuming baseline values as covariates. Effect sizes (ES) were calculated using Cohen’s ES and were interpreted as follows: <0.2, trivial; 0.20 to 0.49, small; 0.50 to 0.80, moderate and >0.80, large [33]. Statistical significance was set at p < 0.05. Statistical analyses were performed by JASP software version 0.10.2 (Amsterdam, Netherlands) for Macintosh.

## Results

Changes in assessment tests before (baseline) and after (post-training) the 6-week intervention period in both groups are shown in Table 3.

**Table 3:** Assessment test before (baseline) and after (post-training) the 6-week intervention period in both groups.

EG						CG					Between group differences	
Variable	Baseline mean ± SD	Post-training mean ± SD	Δ Mean ± SD (95% CI)	*p*	ES	Baseline mean ± SD	Post-training mean ± SD	Δ Mean ± SD (95% CI)	*p*	ES	F	*p*
5-m (s)	1.089 ± 0.068	1.054 ± 0.049	0.036 ± 0.011	0.005	0.770	1.088 ± 0.059	1.071 ± 0.064	0.017 ± 0.015	0.290	0.321	23.619	0.249
10-m (s)	1.903 ± 0.087	1.867 ± 0.087	0.036 ± 0.013	0.016	0.629	1.897 ± 0.093	1.886 ± 0.100	0.012 ± 0.028	0.687	0.119	20.469	0.409
30-m (s)	4.271 ± 0.198	4.256 ± 0.184	0.015 ± 0.022	0.508	0.159	4.382 ± 0.241	4.315 ± 0.208	0.067 ± 0.055	0.252	0.349	39.484	0.721
15m-AG-B (s)	4.832 ± 0.380	4.712 ± 0.358	0.121 ± 0.122	0.338	0.232	4.852 ± 0.308	4.767 ± 0.271	0.085 ± 0.136	0.545	0.181	0.191	0.651
Zigzag-B (s)	7.124 ± 0.656	6.803 ± 0.392	0.321 ± 0.101	0.006	0.747	7.270 ± 0.687	7.169 ± 0.450	0.101 ± 0.084	0.256	0.346	71.742	0.002
T-test (s)	10.507 ± 0.711	9.991 ± 0.440	0.516 ± 0.147	0.003	0.829	10.467 ± 0.767	10.220 ± 0.582	0.247 ± 0.222	0.290	0.321	6.450	0.170
IAT (s)	16.881 ± 1.027	15.983 ± 0.179	0.898 ± 0.242	0.002	0.877	17.029 ± 0.767	16.596 ± 0.805	0.433 ± 0.302	0.179	0.414	0.171	0.005
Arrowhead-R (s)	8.569 ± 0.472	8.199 ± 0.237	0.371 ± 0.092	< 0.001	0.945	8.802 ± 0.514	8.600 ± 0.456	0.202 ± 0.172	0.266	0.338	4.188	0.010
Arrowhead-L (s)	8.732 ± 0.364	8.374 ± 0.284	0.358 ± 0.072	<0.001	1.176	8.671± 0.320	8.554 ± 0.322	0.117 ± 0.086	0.203	0.391	13.154	0.033
NMAT (s)	9.729 ± 0.460	9.378 ± 0.303	0.351 ± 0.093	0.002	0.884	9.352 ± 0.476	9.559 ± 0.435	- 0.208 ± 0.146	0.183	- 0.410	6.863	0.026

EG = Experimental Group; CG = control group; SD = standard deviation; Δ: Real change between pre- and post-training performance; CI = confidence interval; *p* = level of significance; ES = effect size; 5-m = 5 meters sprint test; 10-m = 10 meters sprint test; 30-m = 30 meters sprint test; 15m-AG-B = Agility test 15-m ball dribbling; Zigzag-B = Zig-zag agility tests with ball; IAT = Illinois Agility Test; Arrowhead-R = Arrowhead COD test right; Arrowhead-L = Arrowhead COD test left; NMAT = New Multi-Change of Direction Agility.

### Speed test

In sprinting ability, EG had significant improvements in 5-m (*p* = 0.005; ES = 0.770, moderate) and 10-m (*p* = 0.16; ES = 0.629, moderate), while no significant improvements were observed in 30-m. For CG, no significant differences were observed in linear sprint (i.e., 5-m, 10-m and 30-m). Moreover, between-groups analysis revealed no significant differences in the sprint test.

### Agility tests

In the agility test, EG had significant improvements in in Zigzag-B (*p* = 0.006; ES = 0.747, moderate), T-test (*p* = 0.003; ES = 0.829, large), IAT (*p* = 0.002; ES = 0.877, large), Arrowhead-R (*p* < 0.001; ES = 0.945, large), Arrowhead-L (*p* <0.001; ES = 1.176, large) and NMAT (*p* = 0.002; ES = 0.884, large). For CG, no significant differences were observed. Between-groups analysis revealed differences in favor of the EG in Zigzag-B (F = 71.742; *p* = 0.002), IAT (F = 0.171; *p* = 0.005), Arrowhead-R (F = 4.188; *p* = 0.010), Arrowhead-L (F = 13.154; *p* = 0.033) and NMAT (F = 6.863; *p* = 0.026).

## Discussion

The present study investigated the effects of including ball driving during RST on speed and agility in relation to regular soccer training. The main findings of this study indicate that EG improved in sprint ability (i.e., 5-m and 10-m) and in the agility tests (i.e., Zigzag-B, T-test, IAT, Arrowhead-R, Arrowhead-L and NMAT). However, CG did not show improvements in any variable (5-m, 10-m, 30-m, 15m-AG-B, Zigzag-B, T-test, IAT, Arrowhead-R, Arrowhead-L and NMAT) evaluated. Accordingly, the analysis showed that between-group differences were significant in agility (i.e., Zigzag-B, IAT, Arrowhead-R, Arrowhead-L and NMAT).

RST has been used by many authors as one of the most specific training methods to improve sprinting ability [19,20,23,24], since it mainly uses the muscles involved in the sprinting action [10]. This study’s results are in agreement with other research [19,23] where a vest and sled (i.e., combined resisted sprit) were used as means of training. These authors achieved significant improvements in the first phase of acceleration (0–10 m) in sprinting. Their findings were expected because resistance training (i.e., resisted sprints) is known to improve lower body maximal strength [23,34], which in turn positively affects sprint performance [34]. In addition, training with sled towing is an appropriate method for improving the early acceleration phase of the sprint [10], which explains the importance of horizontal propulsive forces [35]. Nevertheless, in the present study, no significant improvements in 30-m for EG were found. These results are in disagreement with other research where soccer players improved [14,19,24]. The differences between this study’s results and those of previous research could be attributed to a numerous factors, such as competitive level or group training status. Moreover, they may be related to the differences in the overloading the players (i.e., horizontal or vertical oriented) or training load used. For example, in the study by Rey et al. [24], a weighted vest was used as a mean of training, which caused more vertical force orientation, and when running velocity increased during a sprint, the orientation of ground reaction forces changed to a more vertical one [36]. In addition, a recent study has considered different training loads to improve sprint performance [16], highlighting that to improve performance at a distance such as 30-m, the optimal training load should be 40–60% body mass. Therefore, both the orientation (i.e., combined resisted sprint) and the training load (13% body mass) used in the present study could explain the lack of improvement in the 30-m. In relation to the CG, there was no significant improvement in sprinting as in other studies [37]. Standard soccer training should be supplemented by more specific conditional (i.e., strength and power interventions) training.

The EG showed significant improvements in all agility tests, except in 15m-AG-B. It is probable that due to this test’s technical complexity, performance is more dependent on technical than conditional variables [31]. In the other test with the ball, with less complexity (i.e., Zigzag-B), there was a significant improvement. When the ball is included in the RST, the technical demands increase causing possible improvements in actions with the ball, such as: driving, turning or sprinting. This is very important since high-intensity efforts are made during the soccer match with the controlled ball [21]; thus, enhancing technical and conditional skills will improve the match performance of soccer players. However, the CG, who performed standard soccer training (i.e., small sided games, technical and tactical training, etc.), did not significantly improve performance in any agility test. These results are in disagreement with other investigations where, after the application of a small sided games training, the technical skills and performance of soccer players improved [38]. It is true that these authors performed a periodized training of small sided games, while in the present research small sided games were part of the habitual soccer training. In addition, it is possible that the training load in CG was insufficient to improve performance. In this line, and continuing with the agility tests without a ball, the results of this study are in agreement with the findings in other investigations [20,39]. The improvements found are most likely the result of kinetic, kinematic, and neuromuscular adaptations [39] because the current interventions did not contain any decision-making or perceptual stimuli that are determinants of agility [40]. RST training alters biomechanical parameters and horizontal force production adequately enough to improve acceleration [10,39], which may have enhanced COD performance (i.e., agility test) in the current study.

Several limitations should be mentioned here: (1) no unresisted sprint training group was used to compare the effect in relation to EG and CG; (2) the intermediate zone (20-m) was not evaluated in the speed test, where several studies have found significant improvements [14,23]; (3) sprinting with the ball while running was not assessed in a speed test (30–40 m); and, (4) the playing positions of the soccer players were not taken into account. However, it is known that technical and conditional performance is influenced by the different playing positions [41]. Therefore, future studies are needed in order to obtain more evidence about the adaptations caused by the training methods used in the present study, since there is not investigation that introduced the ball in RST.

In practical terms, the present results suggest that strength and conditioning coaches should consider RST as a good method to improve the performance in U-19 elite soccer players, mainly in the early phases of acceleration and in the ability to change direction (i.e., agility tests). Additionally, this study shows a novel and useful finding since the players who did the intervention improved performance in an agility test with the ball. This means that physical trainers can use the ball in conditional exercises to improve not only physical performance but also the technical skills of their players in high-intensity actions (i.e., COD).

## Conclusions

Based on the results hereby presented, the inclusion of the ball driving during the performance of resisted sprints improved sprinting (i.e., 5-m and 10-m) and agility (i.e., T-test, IAT, Arrowhead-R, Arrowhead-L and NMAT), in addition to technical performance (i.e., Zigzag-B). However, CG did not enhance any performance variable. Injury prevention training can reduce injuries in soccer but in this research, it did not improve the performance of soccer players. In conclusion, the standard soccer training should be supplemented with RST, both with ball driving and without ball driving, to improve the performance variables of soccer players.

## Supporting information

S1 File (CSV)
